# Comprehensive management of submandibular salivary duct carcinoma with unilateral axillary lymph node metastasis: a case report

**DOI:** 10.3389/fonc.2026.1814242

**Published:** 2026-05-20

**Authors:** Ning Zhu, Guile Zhao, Qi Han, Yufei Hua, Guanru Wang, Mingzhe Bao, Ning Gao, Chunjie Li

**Affiliations:** 1 Department of Oral and Maxillofacial Surgery, Daqing Oil Field General Hospital, Daqing, China; 2 State Key Laboratory of Oral Diseases & National Center for Stomatology & National Clinical Research Center for Oral Diseases, West China Hospital of Stomatology, Sichuan University, Chengdu, Sichuan, China; 3 Department of Head and Neck Oncology, West China Hospital of Stomatology, Sichuan University, Chengdu, China; 4 Department of Oral Pathology, West China Hospital of Stomatology, Sichuan University, Chengdu, China

**Keywords:** axillary lymph node metastasis, case report, HER2, salivary duct carcinoma, submandibular gland, trastuzumab

## Abstract

**Background:**

Salivary duct carcinoma (SDC) is a rare and aggressive malignancy of the salivary glands, most commonly originating in the parotid gland and less frequently in the submandibular gland. Isolated axillary lymph node metastasis is extremely uncommon, and its clinical characteristics and optimal management remain unclear.

**Case presentation:**

A 69-year-old man presented with a painless swelling in the left submandibular region. Imaging revealed a submandibular mass with ipsilateral cervical and axillary lymphadenopathy, without distant metastasis. Fine-needle aspiration cytology (FNA cytology) supported the diagnosis of salivary duct carcinoma. The patient underwent left submandibular gland resection with radical neck and axillary lymph node dissection. Histopathology revealed poorly differentiated SDC with metastatic deposits in both cervical and axillary nodes. Genetic testing identified HER2 (ERBB2) gene amplification. Postoperatively, the patient received four cycles of combination chemotherapy with nab-paclitaxel and trastuzumab, followed by one year of trastuzumab maintenance therapy. The treatment was well tolerated, and imaging follow-up at 3, 6, and 12 months showed no recurrence or distant metastasis.

**Conclusion:**

Axillary lymph node involvement in submandibular salivary duct carcinoma is extremely rare. To our knowledge, this is the first reported case of isolated unilateral axillary nodal metastasis in the absence of other distant metastases. This case suggests that axillary involvement may represent regional rather than distant spread, and curative surgery remains feasible after comprehensive evaluation. However, this interpretation should be made cautiously because it is based on a single case. Combined chemotherapy and HER2-targeted therapy may achieve favorable short-term outcomes with good tolerability. The accumulation of similar cases is needed to clarify the staging and guide optimal management of such atypical metastases.

## Introduction

Salivary duct carcinoma (SDC) is a rare and highly aggressive epithelial malignancy of the salivary glands, accounting for approximately 1–3% of all salivary gland cancers (1, 2). First described by Kleinsasser et al. in 1968, it closely resembles high-grade mammary ductal carcinoma, and is characterized by cribriform or solid proliferation of atypical ductal cells with comedo-type necrosis (3, 4). It predominantly affects elderly males and most commonly arises in the parotid gland, while the submandibular gland is a less frequent site of origin (5, 6).

Histopathologically, salivary duct carcinoma typically expresses epithelial markers such as cytokeratin, epithelial membrane antigen, and carcinoembryonic antigen (7, 8). In a subset of tumors, overexpression or amplification of HER2 or the androgen receptor (AR) can be detected, offering potential therapeutic targets (9, 10). Despite recent advances, SDC remains an aggressive malignancy with poor prognosis. Radical surgical resection followed by adjuvant chemoradiotherapy is considered the standard treatment, while HER2-targeted therapy with trastuzumab has shown encouraging results in HER2-positive disease (11, 12).

Axillary lymph node metastasis in SDC is exceedingly rare. Anatomical studies reveal lymphatic communication between the submandibular, supraclavicular, and axillary chains via the subclavian lymphatic trunk, suggesting a possible route for atypical lymphatic spread (13). Whether isolated axillary metastasis represents regional extension or distant metastasis remains controversial. Herein, we report what is, to our knowledge, the first documented case of submandibular SDC with isolated unilateral axillary lymph node metastasis in the absence of other distant disease. This case highlights a rare metastatic pattern with potential implications for disease staging, surgical decision-making, and multidisciplinary treatment planning.

## Case presentation

**Figure 1:**
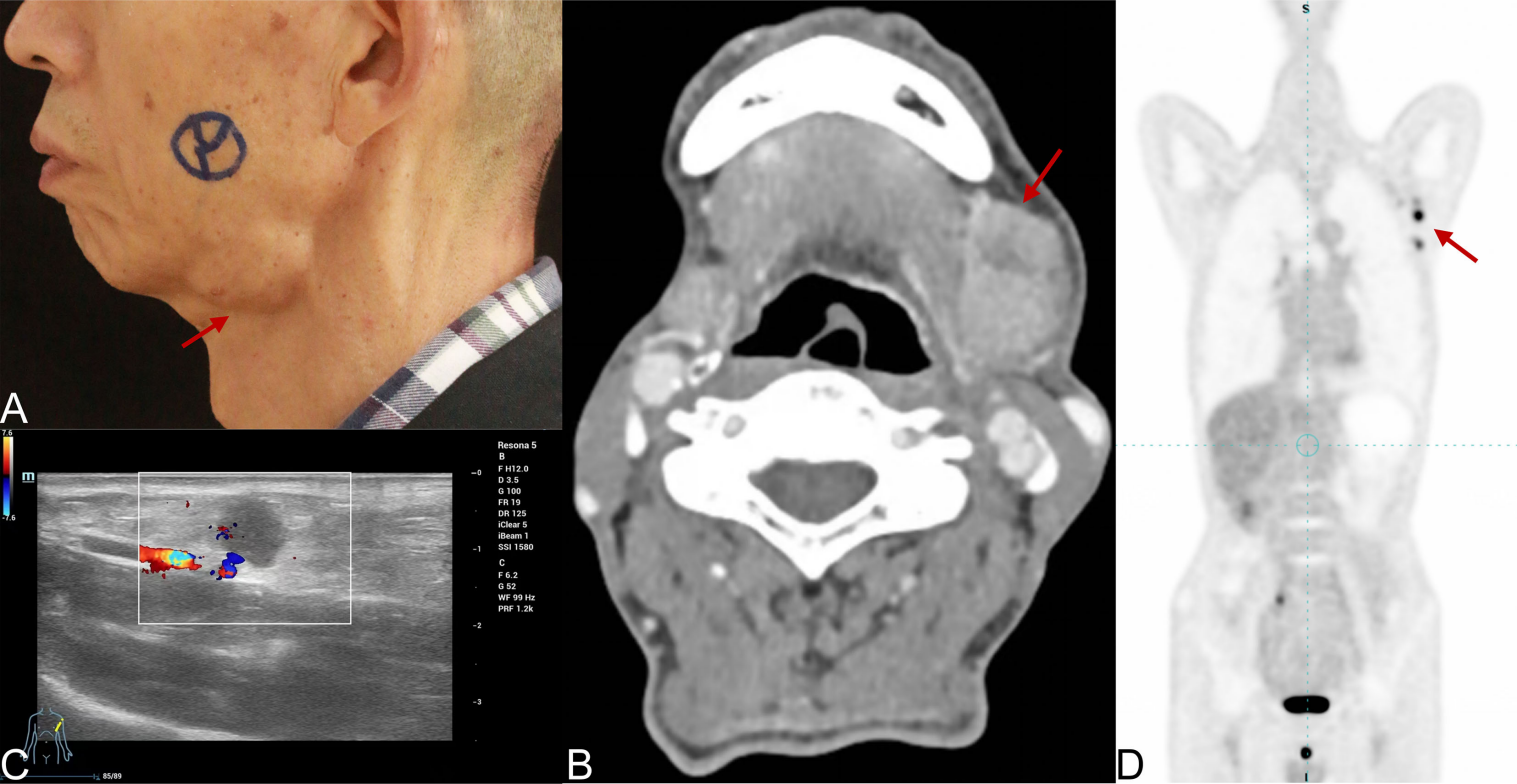
Preoperative clinical and imaging findings of submandibular salivary duct carcinoma with axillary lymph node metastasis. **(A)** Clinical photograph showing a firm mass in the left submandibular region (red arrow). **(B)** Contrast-enhanced CT image revealing an irregular soft-tissue mass with heterogeneous enhancement and central necrosis (red arrow). **(C)** Color Doppler ultrasonography of the left axilla demonstrating a rounded hypoechoic lymph node with blurred cortical–medullary differentiation and increased vascularity (red arrow). **(D)** PET/CT scan showing multiple FDG-avid lesions in the left axillary region (red arrow), indicating metastatic lymphadenopathy.

A 69-year-old man presented with a painless swelling in the left submandibular region for six months, without dysphagia, trismus, or facial nerve dysfunction. He had an ECOG performance status of 0, a history of atrial fibrillation treated with metoprolol succinate, no history of smoking, and a history of alcohol use. Physical examination revealed a firm, fixed mass approximately 4.0 × 3.0 cm below the left mandibular margin, with indistinct borders and a mildly nodular surface (**Figure 1A**). Multiple non-tender, firm lymph nodes were palpable in the left neck and axilla, measuring up to 1.5 cm and 1.2 cm, respectively.

For preoperative evaluation and staging, a multimodality imaging workup was performed, and the detailed acquisition parameters are summarized in **Table 1**. Contrast-enhanced CT demonstrated an irregular soft-tissue mass in the left submandibular region with ill-defined margins and heterogeneous enhancement, containing necrotic areas (**Figure 1B**). Enlarged lymph nodes were noted in the submental and left cervical levels II–IV. Ultrasonography confirmed multiple rounded, hypoechoic lymph nodes in the left axilla with blurred cortical-medullary differentiation and increased vascularity (**Figure 1C**). PET/CT revealed multiple FDG-avid nodes in the submandibular, cervical, and left axillary regions, consistent with metastases (**Figure 1D**). No other distant lesions were detected. Fine-needle aspiration cytology (FNA cytology) of the left submandibular lesion supported the diagnosis of salivary duct carcinoma. Ki-67 immunostaining in the preoperative biopsy specimen showed a proliferative index of approximately 70%. After multidisciplinary evaluation, the disease was considered technically resectable and confined to the primary site, ipsilateral cervical lymph nodes, and unilateral axillary lymph nodes, without evidence of visceral metastasis. Therefore, curative-intent surgery was selected.

**Table 1:** Preoperative imaging and biopsy summary.

Modality	Technical details	Key findings
Contrast-enhanced CT	64-slice multidetector CT scanner; tube voltage 120 kVp; automated tube current modulation 150–250 mAs; slice thickness 1.25 mm; reconstruction interval 1.0 mm; iohexol 300 mgI/mL, 80–100 mL; injection rate 2.0–3.0 mL/s; venous phase at 60–70 s.	Irregular soft-tissue mass in the left submandibular region with ill-defined margins, heterogeneous enhancement, and necrotic areas; enlarged lymph nodes in the submental and left cervical levels II–IV.
Axillary ultrasonography	Bilateral axillary ultrasonography performed to assess clinically palpable left axillary nodes.	Multiple rounded, hypoechoic lymph nodes in the left axilla with blurred cortical-medullary differentiation and increased vascularity.
^18^F-FDG PET/CT	Fasting state; blood glucose 5.80 mmol/L; 18F-FDG dose 5.65 mCi; image acquisition 60 min after injection	Hypermetabolic left submandibular lesion measuring approximately 2.9 × 2.0 × 3.8 cm (SUVmax 16.2); multiple FDG-avid lymph nodes in the submental, left submandibular, cervical, and left axillary regions; the largest suspicious lymph node in the submental region measured 1.5 × 1.3 cm (SUVmax 11.0); no other definite distant metastatic lesions identified
Ultrasound-guided fine-needle aspiration biopsy	22-gauge needle; 3 passes were performed under ultrasound guidance, and the obtained material was submitted for pathological evaluation according to institutional protocol.	Histopathological findings were consistent with salivary duct carcinoma.

### Surgery

**Figure 2:**
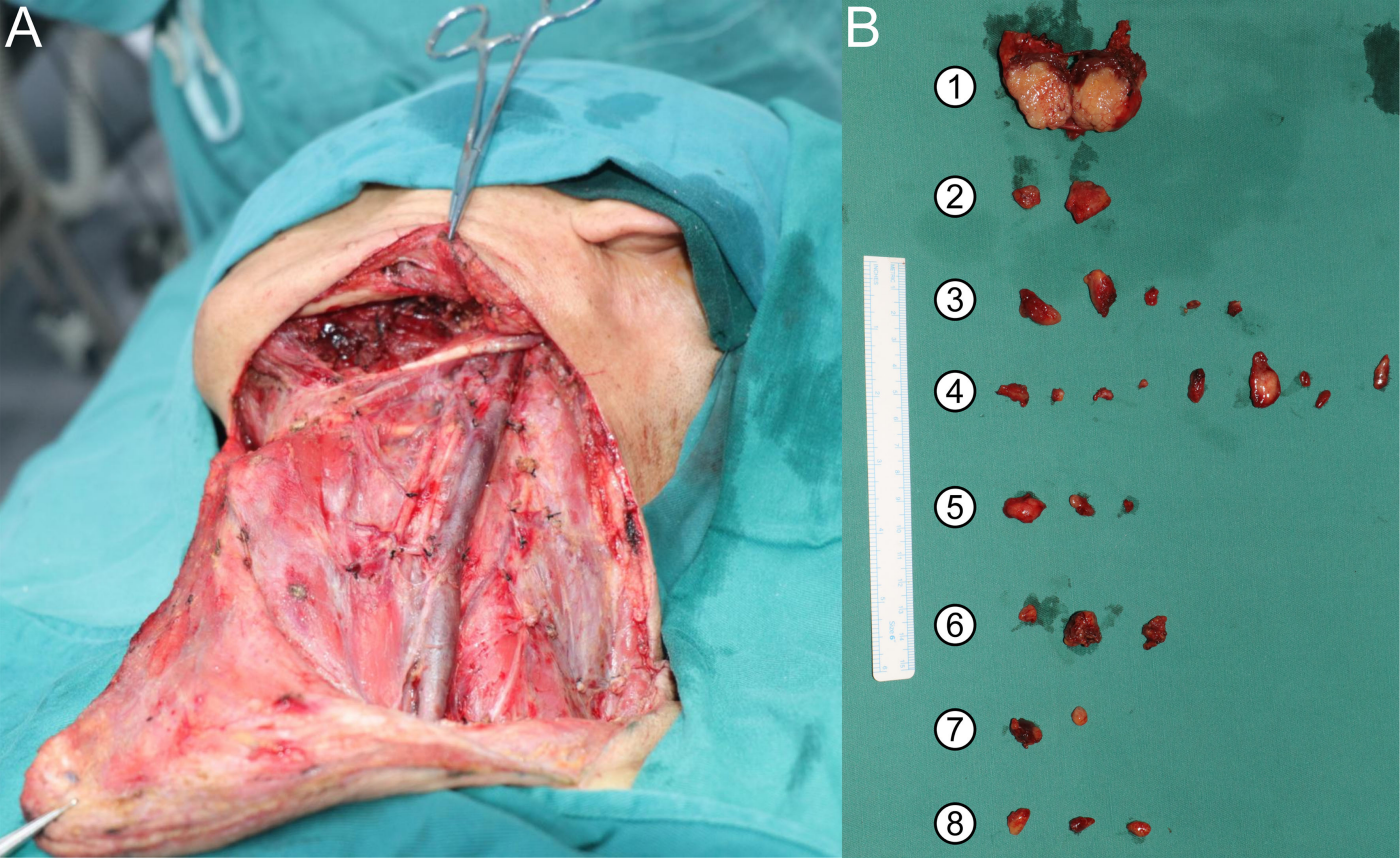
Intraoperative findings and resected specimens of submandibular salivary duct carcinoma with axillary lymph node metastasis. **(A)** Intraoperative view showing exposure of the left submandibular region after gland resection and radical neck dissection. **(B)** Resected specimens from different anatomical regions. Numbers indicate: the bisected primary submandibular tumor; submental lymph nodes; submandibular lymph nodes; upper deep cervical lymph nodes; middle deep cervical lymph nodes; lower deep cervical lymph nodes; axillary lymph nodes.

Under general anesthesia, the patient underwent left submandibular gland resection with radical neck dissection and left axillary lymph node dissection involving levels I–III (**Figure 2A**). Intraoperatively, a firm, gray-white mass with focal necrosis was observed in the left submandibular region (**Figure 2B**).

### Pathology

**Figure 3:**
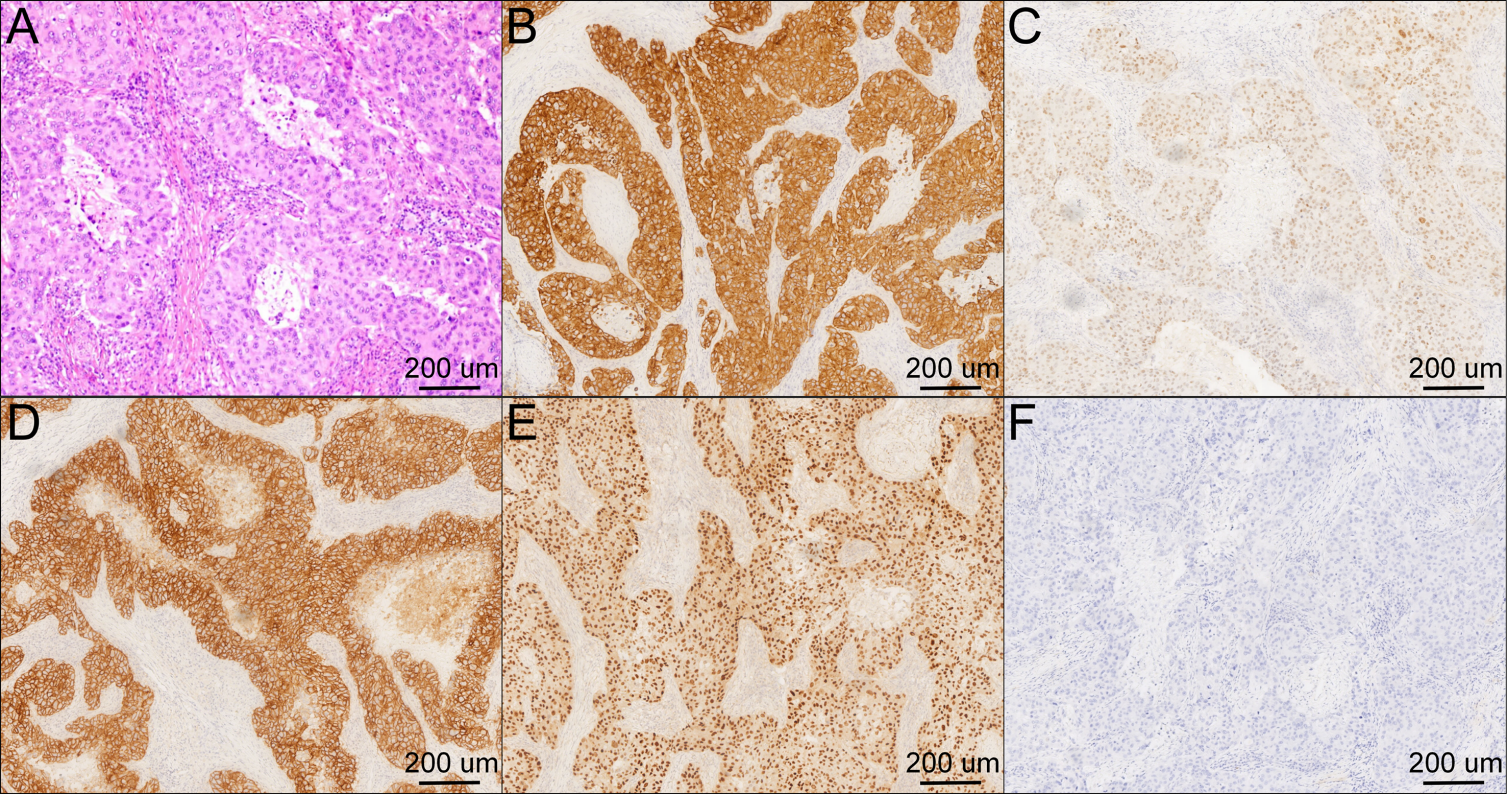
Histopathological and immunohistochemical features of submandibular salivary duct carcinoma. **(A)** Hematoxylin and eosin (H&E) staining showing solid and cribriform proliferation of atypical ductal epithelial cells with comedo-type necrosis and fibrous stromal infiltration. **(B–F)** Immunohistochemical staining revealing strong positivity for CK7 **(B)**, focal nuclear expression of P53 **(C)**, intense membranous HER2 staining **(D)**, positive androgen receptor (AR) expression **(E)**, and negative S-100 **(F)**. Original magnification ×200 for all panels. Scale bars = 200 µm.

Histopathology revealed a poorly differentiated salivary duct carcinoma with metastatic deposits in both cervical and axillary lymph nodes (**Figure 3A**). Detailed pathological nodal mapping is summarized in **Table 2**. Immunohistochemical analysis was performed on formalin-fixed, paraffin-embedded tissue sections using an automated staining platform according to institutional diagnostic protocols. Detailed antibodies, clones, dilutions, staining results, and interpretation criteria are summarized in **Table 3**. Immunohistochemistry showed CK7 (+), P16 (+), P53 (focal +), AR (+), P63 (focal +), Vimentin (+), and S-100 (−). HER2 showed strong complete membranous staining and was scored as 3+ (**Figures 3B–F**). According to the AJCC 8th edition TNM staging system, the postoperative pathological stage was pT2N2bM1.

**Table 2:** Pathological nodal mapping and metastatic status.

Dissected compartment	Positive / examined	Largest metastatic lymph node	Extranodal extension
Submental	2/2	<3 cm	Negative
Submandibular	2/5	<3 cm	Negative
Cervical level II	2/9	<3 cm	Negative
Cervical level III	2/3	<3 cm	Negative
Cervical level IV	2/3	<3 cm	Negative
Supra-axillary compartment	1/2	<3 cm	Negative
Inferior axillary compartment	1/3	<3 cm	Negative

No distinct lymph node was separated from the central axillary compartment. All metastatic lymph nodes measured <3 cm in greatest dimension, and no extranodal extension was identified.

**Table 3:** Immunohistochemical methods and results.

Marker	Clone	Dilution	Platform	Staining pattern / result	Interpretation / scoring criteria
CK7	OV-TL 12/30	1:200	BenchMark Ultra	Diffuse cytoplasmic positive	Supports ductal epithelial differentiation
P16	E6H4	Prediluted	BenchMark Ultra	Diffuse nuclear and cytoplasmic positive	Diffuse nuclear and cytoplasmic staining interpreted as positive
P53	DO-7	1:100	BenchMark Ultra	Focal nuclear positive	Focal nuclear staining; significance interpreted in the overall histopathological context
HER2	4B5	Prediluted	BenchMark Ultra	Strong circumferential membranous positive (3+)	Scored according to the 2018 ASCO/CAP breast cancer criteria; 3+ defined as strong complete membranous staining in >10% of tumor cells
AR	AR441	1:100	BenchMark Ultra	Diffuse nuclear positive	Positive defined as nuclear staining in >10% of tumor cells
P63	4A4	1:400	BenchMark Ultra	Focal nuclear positive	Focal nuclear positivity
Vimentin	V9	1:100	BenchMark Ultra	Diffuse cytoplasmic positive	Diffuse cytoplasmic positivity
S-100	Polyclonal	1:2500	BenchMark Ultra	Negative	Negative; argues against myoepithelial or neural differentiation

### Postoperative treatment and follow-up

The patient recovered uneventfully after surgery, with no wound infection or functional impairment. Targeted next-generation sequencing was performed on the resected tumor and matched blood sample, which confirmed ERBB2 amplification (copy number 13.4) and identified a TP53 c.981T>A (p.Y327*) variant (variant allele frequency 40.3%). Based on published evidence supporting trastuzumab-taxane regimens in HER2-positive salivary duct carcinoma, a regimen of nab-paclitaxel plus trastuzumab was selected (12, 14, 15). A peripherally inserted central catheter (PICC) was placed before treatment initiation. One month postoperatively, combination therapy with nab-paclitaxel (200 mg, day 1) plus trastuzumab (loading dose 8 mg/kg on cycle 1 day 1, followed by 6 mg/kg every 3 weeks) was administered for four cycles, followed by maintenance trastuzumab monotherapy (6 mg/kg every 3 weeks) for one year. Postoperative radiotherapy was not administered. Supportive care included prophylactic antiemetic therapy, gastric mucosal protection, and intravenous hydration according to institutional protocols.

Before initiating trastuzumab, baseline cardiac assessment was performed, including electrocardiography and transthoracic echocardiography. Electrocardiography demonstrated atrial fibrillation, and echocardiography showed left atrial enlargement with mild-to-moderate mitral regurgitation; left ventricular systolic function was preserved. Given the pre-existing cardiac condition, cardiac surveillance was particularly emphasized, with follow-up echocardiography scheduled every two cycles according to institutional clinical practice. The treatment was generally well tolerated, with grade 1 fatigue and grade 1 alopecia according to CTCAE criteria and no serious adverse events. No treatment delay or dose reduction was required. Postoperative surveillance consisted of clinical evaluation, contrast-enhanced head and neck CT, and chest CT at 3, 6, and 12 months after surgery, which showed no evidence of local recurrence or distant metastasis (**Table 4**).

**Table 4:** Timeline.

Time	Clinical event
6 months before admission	The patient noticed a painless swelling in the left submandibular region, which gradually enlarged.
At admission	Physical examination revealed a firm 4.0 × 3.0 cm mass in the left submandibular region with palpable cervical and axillary lymphadenopathy.
Preoperative evaluation	Contrast-enhanced CT, ultrasonography, and PET-CT demonstrated a submandibular mass with ipsilateral cervical and left axillary lymph node metastases; no distant metastasis was identified. Fine-needle aspiration and biopsy confirmed salivary duct carcinoma.
Surgery	The patient underwent left submandibular gland resection with radical neck dissection and left axillary lymph node dissection.
Postoperative pathology	Histopathology confirmed poorly differentiated salivary duct carcinoma with metastatic deposits in both cervical and axillary lymph nodes. Immunohistochemistry showed HER2 positivity, and genetic testing confirmed ERBB2 gene amplification.
1 month after surgery	Four cycles of combination therapy with nab-paclitaxel and trastuzumab (every 3 weeks) were initiated.
Months 2–5 after surgery	Completion of four cycles of chemotherapy combined with trastuzumab.
Months 6–18 after surgery	Maintenance trastuzumab monotherapy was administered for one year.
3, 6, and 12 months follow-up	Imaging follow-up showed no evidence of local recurrence or distant metastasis.

## Discussion

This report describes a rare case of submandibular SDC with isolated axillary lymph node metastasis. Axillary involvement in salivary gland malignancies is extremely uncommon. A literature review revealed that SDC typically metastasizes to the cervical lymph nodes, lungs, bones, and liver, with axillary metastasis being exceptionally rare (1, 16). A review of the available literature identified only a small number of reported cases of axillary metastasis from salivary gland primary tumors, including both salivary duct carcinoma and oncocytic carcinoma, and these cases are summarized in **Table 5** (17–20). Most previously reported cases arose from the parotid gland and were associated with more extensive locoregional or distant metastatic disease. In contrast, the present patient had submandibular SDC with isolated unilateral axillary lymph node metastasis and no evidence of visceral or other distant metastatic disease. To our knowledge, this represents the first reported submandibular SDC with this metastatic pattern. This case therefore expands the currently recognized spectrum of metastatic spread in SDC and provides additional clinical context for interpreting unusual nodal dissemination.

**Table 5:** Previously reported cases of axillary metastasis from salivary gland primary tumors.

Author	Year	Primary site/diagnosis	Metastatic sites	Treatment	HER2/AR status	Outcome
Khetan et al. (17)	2026	left parotid gland/salivary duct carcinoma	Cervical and axillary lymph nodes; diffuse left neck soft tissue/skin involvement	US-FNA–based diagnosis; paclitaxel + carboplatin + pembrolizumab	HER2 negative; AR positive	Improvement in neck induration after 4 cycles; transferred to another facility for further care
Stefanovski et al. (18)	2026	Parotid gland/oncocytic carcinoma	Cervical, supraclavicular, mediastinal, hilar, interpectoral, left brachial, and bilateral axillary lymph nodes; thyroid; left pectoral muscle; subcutaneous and skin metastases	Radical surgery (parotidectomy + neck dissection); adjuvant radiotherapy; pembrolizumab; later chemotherapy; then palliative/supportive care	Not reported	Progressive metastatic disease; died 4 years after diagnosis
Krishnamurthy et al. (19)	2013	Parotid gland/salivary duct carcinoma	Contralateral cervical lymph nodes; lung metastasis; mediastinal lymph nodes	Parotidectomy + modified radical neck dissection; postoperative chemoradiation; contralateral neck dissection; radiotherapy to neck and axilla; carboplatin; docetaxel + trastuzumab followed by maintenance trastuzumab	HER2 3+; AR not reported	Partial response to trastuzumab-based therapy; no progression 9 months after discontinuation; alive and clinically well at later follow-up
Chhabra et al. (20)	2012	Lip minor salivary gland/oncocytic carcinoma	Bilateral submandibular lymph nodes; right axillary lymph node metastasis	Surgery; FNA confirmation of nodal metastases	Not reported	Follow-up outcome not clearly reported

The mechanism of axillary spread remains unclear. Anatomical studies suggest lymphatic communication between the submandibular, supraclavicular, and axillary chains via the subclavian lymphatic trunk, allowing “skip” metastasis beyond conventional cervical drainage pathways (13). Preoperatively, the main differential considerations included atypical regional lymphatic spread from the submandibular primary, true distant metastatic disease, hematogenous dissemination, and the possibility of a synchronous occult primary malignancy associated with axillary nodal involvement. Among these possibilities, atypical lymphatic spread was considered the most plausible explanation in this case because the metastatic burden was confined to the ipsilateral cervical and axillary nodal chains, without evidence of visceral or other distant metastatic disease, and no alternative primary lesion was identified on preoperative imaging. This distribution pattern was less suggestive of hematogenous dissemination, which more commonly involves distant organs. The classification of this pattern within the TNM framework remains uncertain. If interpreted as distant metastasis (M1), the disease would generally be managed as systemic metastatic disease, and curative-intent surgery might be considered less appropriate. However, if regarded as atypical regional lymphatic extension, surgical resection may still be justified in carefully selected patients. In the present case, the primary lesion and all radiologically identified nodal disease were anatomically resectable, and no visceral or other distant metastatic lesions were detected. Therefore, multidisciplinary evaluation favored combined radical neck and axillary dissection as a curative-intent approach rather than upfront systemic therapy. Nevertheless, this interpretation should be made cautiously, as this single case cannot definitively establish whether axillary nodal involvement represents regional extension or true distant spread. The favorable short-term postoperative outcome further supports the feasibility of this approach in carefully selected patients.

Beyond surgical decision-making, molecular profiling also informed postoperative systemic therapy. HER2-positive SDCs have shown high response rates to trastuzumab combined with taxane-based chemotherapy (21). In this case, the patient with confirmed HER2 amplification achieved excellent disease control and tolerance with nab-paclitaxel plus trastuzumab, followed by one year of maintenance trastuzumab therapy. This underscores the therapeutic value of molecularly guided treatment in rare metastatic SDCs.

## Conclusion

Isolated axillary lymph node metastasis in submandibular SDC may, in selected cases, represent atypical regional lymphatic spread rather than unequivocal distant metastasis. However, conclusions regarding staging and prognosis should be drawn cautiously because this report describes only a single case with limited follow-up. Comprehensive evaluation and multidisciplinary management, together with HER2-targeted therapy, may provide favorable short-term outcomes in such uncommon presentations. Further accumulation of similar cases is warranted to clarify the staging implications of axillary involvement and to optimize integrated treatment strategies.

## Data Availability

The raw data supporting the conclusions of this article will be made available by the authors, without undue reservation.
